# Supplementary material for: Putative Daucus carota Capsanthin-Capsorubin Synthase (DcCCS) Possesses Lycopene β-Cyclase Activity, Boosts Carotenoid Levels, and Increases Salt Tolerance in Heterologous Plants
Source: Plants (Basel). 2023 Jul 27;12(15):2788. doi: 10.3390/plants12152788 (PMC10421225; doi:10.3390/plants12152788)
Supplement: Supplementary file 1 [file plants-12-02788-s001.zip › plants-2492568-supplementary.pdf]

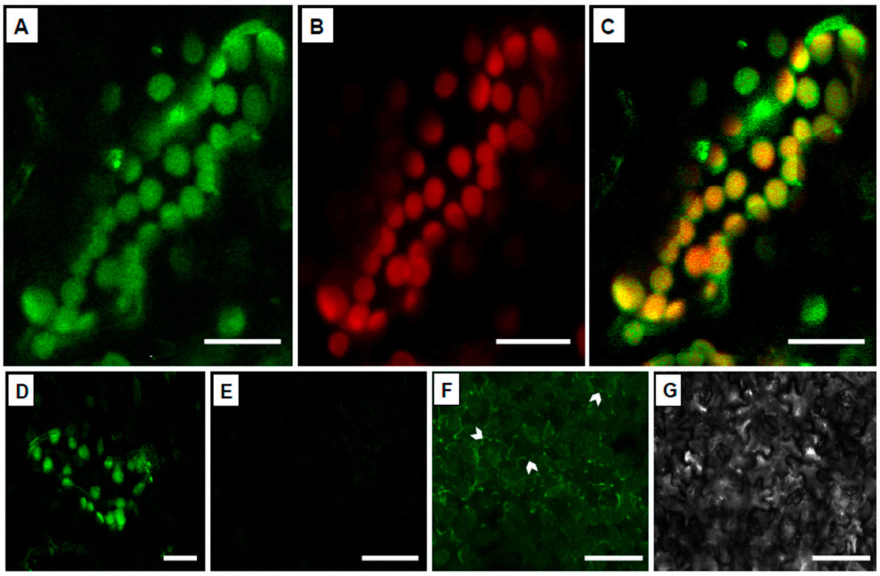

Figure S1. Subcellular localization of the DcLCYB2:GFP **A)** Fluorescence of epidermal cells from two-month-old tobacco leaves that were agroinfiltrated with *A. tumefaciens* transformed with the pGWB5/DcLCYB2:GFP. After 4 days, explants were observed by confocal fluorescence microscopy, where a punctate fluorescence pattern was detected from discrete structures indicative of the chloroplastic localization of DcLCYB2:GFP. **B)** Autofluorescence emission of chlorophyll contained in the plastids of agroinfiltrated tobacco cells. **C)** Colocalization of the fluorescence signals detected in A and B. **D.** Positive control: Fluorescence of tobacco epidermal cells expressing recA:YFP. **E)** Leaf tissue infiltrated with agroinfiltration medium (negative control). **F)** Fluorescence of tobacco epidermal cells transformed with pGWB5 empty vector (negative control), indicating the cytoplasmic localization of soluble GFP. The white arrowheads show the plasma membrane of the cell. **G)** Phase contrast image indicating the morphology and boundaries of the transformed tobacco epidermal cells. Images were captured with a 40X (A-C) and 20X (D-G) magnification. Fluorescence was observed after excitation at 489 nm. Scale bar: 20µm (A-D) and 100µm (E-G).

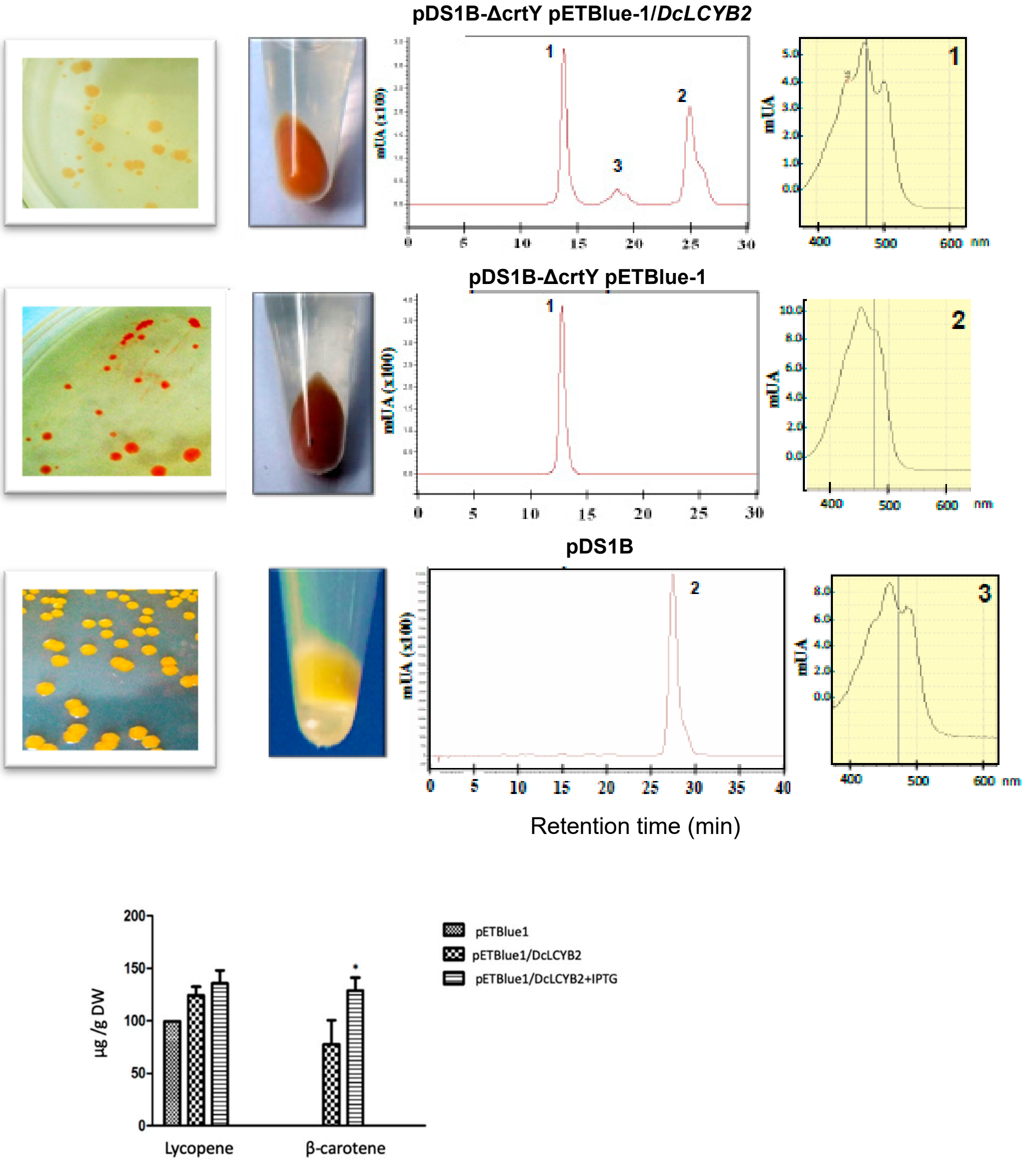

Figure S2 Quantification of carotenoids produced in *E. coli* BL21 strain complemented with *DcLCYB2* Carotenoids were extracted from liquid bacterial BL21 cultures harboring pDS1BΔcrtY and transformed with either pET-Blue1/LCYB2, pET-Blue1(negative control) or pDS1B (positive control). The bacterial colonies and pellet from each transformed strains after complementation are shown in boxes at left of each chromatogram. Chromatograms show that both lycopene (peak 1) and β-carotene (peak 2) are present in the strain transformed with DcLcyb2, while the control was not able to restore the mutation of crtY gene in the strain, producing only lycopene. The spectra of lycopene and β-carotene are shown in the right side of the figure with number 1 and 2, respectively. Peak 3 corresponds to the intermediate y-carotene (Britton et al., 2008). The lycopene and β-carotene content were quantified and shown in the lower graph. Asterisks indicate significant differences determined by tailed paired T-test (p<0.05).

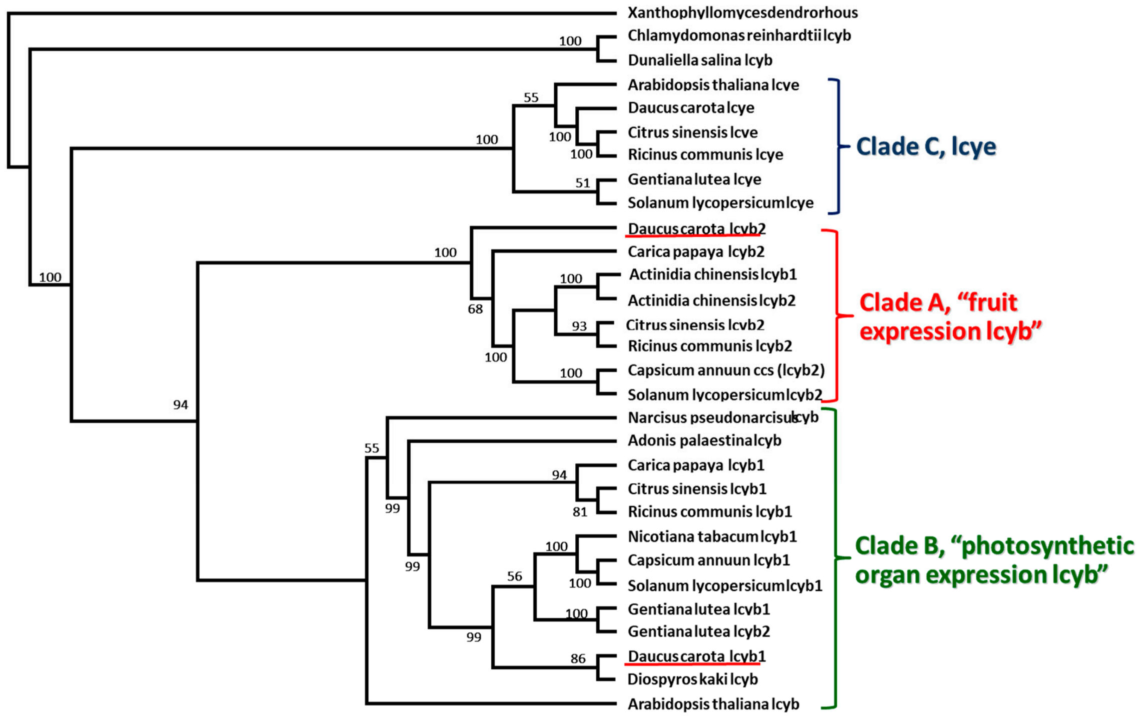

Figure S3 Phylogenetic tree based on nucleotide sequences of lycopene cyclases enzymes. The phylogenetic tree was built using the Neighbor-joining method. Numbers adjacent to branches are bootstrap values supporting this tree. CRTY (LCYB) from *Xhantophyllomyces dendrorhous* was used for rooting. The different lycopene cyclase genes are associated in three different clades A: clade associated with fruit expression B: clade associated with photosynthetic organ expression, and C: clade associated with *lcyE*.

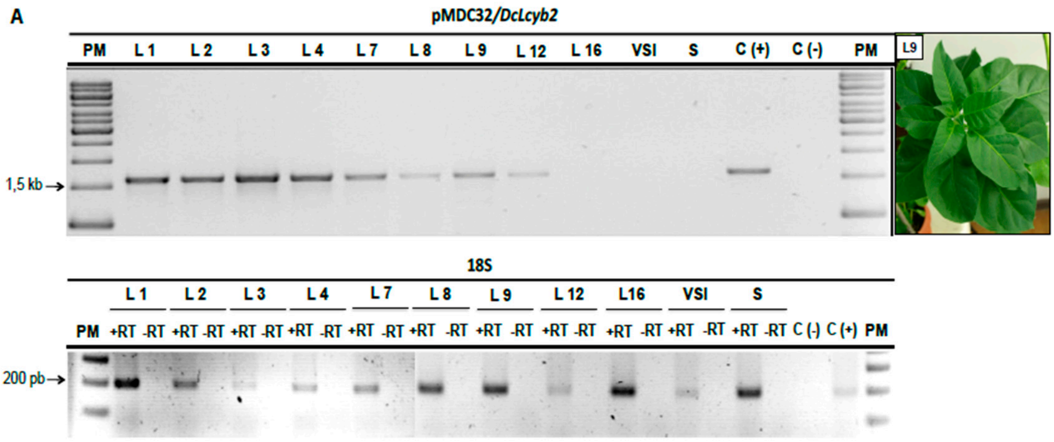

Figure S4 Identification of independently transformed tobacco lines with pMDC32/DcLCYB2. **A**. RT-PCR analysis of tobacco lines transformed with the pMDC32/DcLCYB2 construct. *DcLCYB2* amplified a 1612 bp fragment. **B**. amplification of the rRNA18s shows the cDNA integrity (+RT, Reverse Transcriptase) and the absence of gDNA contamination (-RT). All lines except L16 harbored the *DcLCYB2*. The representative image of leaves from L9 used for molecular analyses is shown on the right. VSI: cDNA template from tobacco leaves transformed with the empty vector pMDC32. S: cDNA samples from wild tobacco leaves. C(-): No template, PCR Negative control. C(+): Positive control from pMDC32/DcLCYB2 pDNA. PM: 1 kb Molecular weight marker.

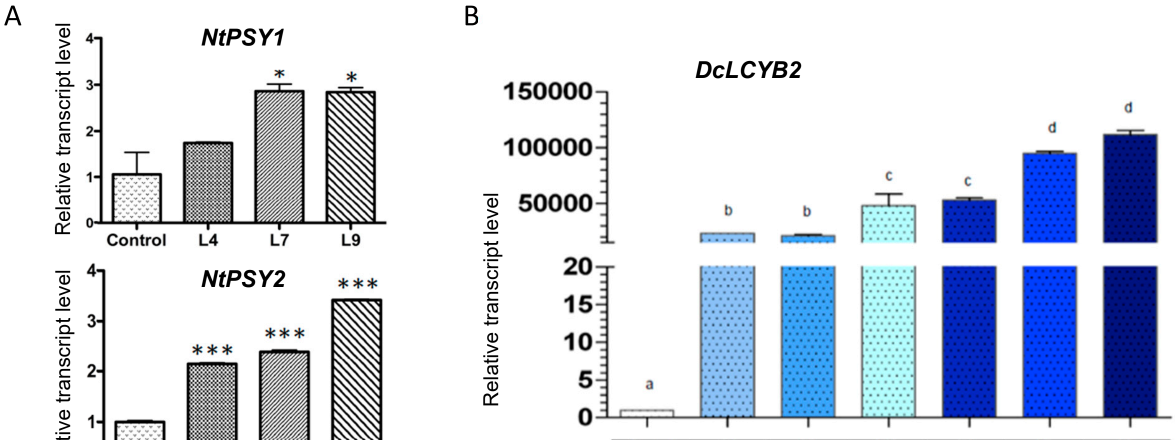

	Control	L1	L8	L12	L4	L7	L9
β-caroteno (µg/g PS)	345.6 ± 48.02	461.0 ± 169.6	554.1 ± 49.21	422.8 ± 12.36	634.2 ± 163.5	600.2 ± 39.19	649.6 ± 43.31

Figure S5 Relative expression of *NtPSY1, NtPSY2* and *DcLCYB2* in tobacco lines transformed with pMDC32/*DcLCYB2*.

Transcript levels of A) endogenous *NtPSY1, NtPSY2* and B) *DcLCYB2* in T0 transgenic tobacco lines transformed with pMDC32/DcLCYB2. The values correspond to technical duplicates of two biological replicates for each line, normalized to the expression of the rRNA18S gene. β-carotene levels previously detected for each line are tabulated beneath each line. *NtPSY1, NtPSY2* expression was determined in lines that presented the highest β-carotene levels. A) Statistical analysis was carried by a two-tailed unpaired T-test.. *: p<0,05 **: p<0,01 *** p<0,001. B) Letters denote significant differences between the expression averages of each line compared to the other lines in the analysis (one-way ANOVA. Post-test Bonferroni).

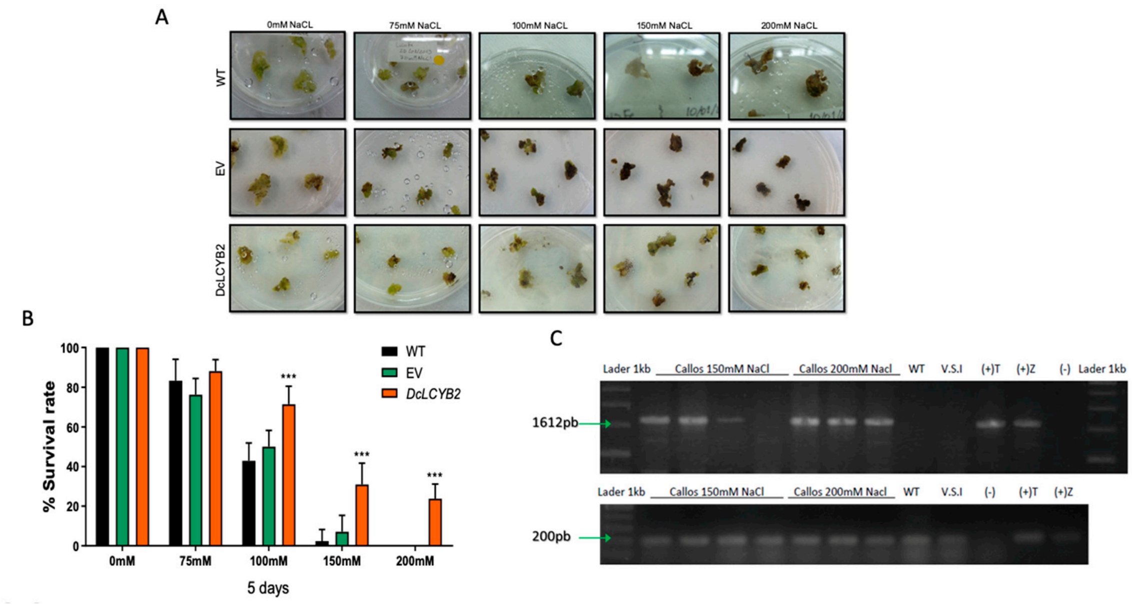

Figure S6. *A. deliciosa* calli survival curve. A) Phenotypic analysis of *DcLCYB2* transformant and WT *A. deliciosa* calli after 5 days under chronic salt treatment (0mM, 75mM, 100mM, 150mM and 200mM NaCl). B) Survival rate of *DcLCYB2* transformant and WT *A. deliciosa* calli after 5 days under chronic salt treatment with different concentrations of NaCl (0mM, 75mM, 100mM, 150mM and 200mM NaCl). C) Representative RT-PCR for *DcLCYB2* in survival calli after 15-day chronic salt treatment. All values represent the means of three independent replicates (+SD). Statistically significant differences were determined by one-tailed ANOVA test and Bonferroni post-test: * $p < 0.05$, ** $p < 0.01$, *** $p < 0.001$. WT: Wildtype; EV: Empty vector. (-): Negative water *DcLCYB2* RT-PCR control; (-)Ad: Wildtype *A. deliciosa* negative *DcLCYB2* RT-PCR control; (+)Nt: DcLCYB2 transgenic tobacco positive RT-PCR control; (+)Dc: wildtype *D. carota* positive *DcLCYB2* RT-PCR control; L1kb: 1kb ladder; L100: 100bp ladder; bp: base pair.

Table S1. List of primers used in this study.

Primer	Gene	Sequence (5´- 3´)	Purpose
DcLycb2_F		5'- ATG GAG ACC CTT AAA TTT ATC AG-3	To amplify the complete cDNA of *DcLCYB2* for ectopic plant expression and complementation
DcLycb2_R		5'- GAT AAT ACA TGG TTA CAA ACA TTG -3'	
DcLycb2_Fq		5'- GCT TGC GAT GCT TAG CTT GTC ACT C-3'	To measure the expression of *DcLCYB2* by qRT
DcLycb2_Rq		5'- CCA TAC CAC CAT TCA CCA ACT CCC-3'	
DcLycb2_R-nst	*DcLCYB2*	5'- GAT AAT ACA TGG TTA CAA ACA -3'	To amplify *DcLCYB2* with DcLycb2_F for sub-cellular localization
DcLycb1_Fq		5'-TGAGTGCAGCTTACACCTACTTGATTA-3'	
DcLycb1_Rq	*DcLCYB1*	5' AACTGCAGAAGATATTGGAGA-3'	To measure the expression of *DcLCYB1* by qRT
DcUbiFq		5-GCTCGAGGACGGCAGAAC-3	
DcUbiRq	*DcUbi*	5-CTTGGGCTTGGTGTAGGTCTTC-3	To measure the expression of *DcUbi* by qRT, housekeeping gene
NtPSY1_Fq		5'-GGA ACC AAG CTA ATG ACC CCA GAG A-'3	
NtPSY1_Rq	*NtPSY1*	5'-GGC CGC CCA CTG AAA ATA TCT TCC-3'	To measure the expression of *NtPSY1* by qRT
NtPSY2_Fq		5'-TCA GAG ATG TAG GAG AAG ATG C-'3	To measure the expression of *NtPSY2* by qRT
NtPSY2_Rq	*NtPSY2*	5'-GCT TCA ATC TCG TCC AAT ATC TTG-3	
NtRNAr18SFq		5'-TTGATTACGTCCCTGCCCTTT-3'	To measure the expression of *NtRNAr18S* by qRT, housekeeping gene
NtRNAr18SRq	RNAr18S	5-' ACAATGATCCTTCCGCAGGT-3'	
